# Glutathione metabolism in the prefrontal brain of adults with high-functioning autism spectrum disorder: an MRS study

**DOI:** 10.1186/s13229-017-0122-3

**Published:** 2017-03-07

**Authors:** Dominique Endres, Ludger Tebartz van Elst, Simon A. Meyer, Bernd Feige, Kathrin Nickel, Anna Bubl, Andreas Riedel, Dieter Ebert, Thomas Lange, Volkmar Glauche, Monica Biscaldi, Alexandra Philipsen, Simon J. Maier, Evgeniy Perlov

**Affiliations:** 1 Section for Experimental Neuropsychiatry, Department of Psychiatry, Medical Center University of Freiburg, Faculty of Medicine, University of Freiburg, Hauptstr. 5, 79104 Freiburg, Germany; 2 Department for Psychiatry and Psychotherapy, Saarland University Medical Center, Kirrberger Str. 100, 66421 Homburg, Saar Germany; 3 Department of Radiology, Medical Physics, Medical Center University of Freiburg, Faculty of Medicine, University of Freiburg, Breisacher Str. 60a, 79106 Freiburg, Germany; 4 Department of Neurology, Medical Center University of Freiburg, Faculty of Medicine, University of Freiburg, Breisacher Str. 64, 79106 Freiburg, Germany; 5 Department for Child and Adolescent Psychiatry and Psychotherapy, Medical Center University of Freiburg, Faculty of Medicine, University of Freiburg, Hauptstr. 8, 79104 Freiburg, Germany; 6 School of Medicine and Health Sciences, Psychiatry and Psychotherapy - University Hospital, Karl-Jaspers-Klinik, Medical Campus University of Oldenburg, Hermann-Ehlers-Str. 7, 26160 Bad Zwischenahn, Germany; 7 Clinic for Psychiatry Luzern, Schafmattstrasse 1, 4915 St. Urban, Switzerland

**Keywords:** Autism spectrum disorder, Asperger syndrome, MR spectroscopy, Glutathione, Anterior cingulate cortex, DLPFC

## Abstract

**Background:**

Autism spectrum disorder (ASD) is a neurodevelopmental disease characterized by difficulties in social communication, unusually restricted, repetitive behavior and interests, and specific abnormalities in language and perception. The precise etiology of ASD is still unknown and probably heterogeneous. In a subgroup of patients, toxic environmental exposure might lead to an imbalance between oxidative stress and anti-oxidant systems. Previous serum and postmortem studies measuring levels of glutathione (GSH), the main cellular free radical scavenger in the brain, have supported the hypothesis that this compound might play a role in the pathophysiology of autism.

**Methods:**

Using the method of single-voxel proton magnetic resonance spectroscopy (MRS), we analyzed the GSH signal in the dorsal anterior cingulate cortex (dACC) and the dorsolateral prefrontal cortex (DLPFC) of 24 ASD patients with normal or above average IQs and 18 matched control subjects. We hypothesized that we would find decreased GSH concentrations in both regions.

**Results:**

We did not find overall group differences in neurometabolites including GSH, neither in the dorsal ACC (Wilks’ lambda test; *p* = 0.429) nor in the DLPFC (*p* = 0.288). In the dACC, we found a trend for decreased GSH signals in ASD patients (*p* = 0.076).

**Conclusions:**

We were unable to confirm our working hypothesis regarding decreased GSH concentrations in the ASD group. Further studies combining MRS, serum, and cerebrospinal fluid measurements of GSH metabolism including other regions of interest or even whole brain spectroscopy are needed.

## Background

### Autism spectrum disorder (ASD)

ASD is a neurodevelopmental disorder characterized by difficulties in social communication and unusually restricted, repetitive behavior and interests with a strong desire for routines [1, 2]. In addition, there are specific abnormalities in language, sensory, and social perception [2]. ASD is an etiologically and phenotypically heterogeneous disorder [3]. In daily clinical practice, phenotypes with subnormal and normal/above average intelligence are distinguished [2, 4]. While the International Classification of Diseases, tenth edition (ICD-10) distinguishes childhood autism (F84.0), atypical autism (F84.1), and Asperger syndrome (ICD-10 F84.5) (www.dimdi.de/static/de/klassi/icd-10-gm/kodesuche/onlinefassungen/htmlgm2015/block-f80-f89.htm), in the current fifth version of the Diagnostic and Statistical Manual of Mental Disorders (DSM-5), these categories are summarized into a single category called ASD (299.00) (www.dsm5.org). Prevalence figures for ASD vary between 1 and 2%, with increased incidence in males [5, 6]. ASD is associated with high prevalence rates of comorbid classical psychiatric disorders such as depression, anxiety, attention deficit hyperactivity disorder (ADHD), psychotic symptoms, or emotionally unstable syndromes [7, 8]. The increasing importance of ASD is caused by the high prevalence rates, the fact that ASD is a life-long condition, and the high rates of psychiatric comorbidity [2].

### GSH and etiology of ASD

The precise etiology of ASD is still unknown and probably heterogeneous [1, 2, 9, 10]. Genetic aspects play a certain role in ASD [2, 11]. Moreover, ASD may develop as a consequence of acquired central nervous system diseases, such as traumatic brain injury with epilepsy or encephalitis [2, 4]. We earlier suggested an excitatory/inhibitory imbalance. Following this hypothesis, a disturbed equilibrium between the most important excitatory neurotransmitter glutamate and the most important inhibitory transmitter γ-amino-butyric acid (GABA) might destabilize cortical networks, which in turn are related to autistic symptoms [9,10]. Another pathophysiological hypothesis of autism holds that an imbalance between oxidative stress and antioxidant systems (in particular glutathione [GSH], the main cellular free radical scavenger in the brain), triggered by toxic environmental exposures, might play a central role in autism. The so-called redox/methylation hypothesis is able to bring different findings in ASD together (“A ‘unified field theory’ of autism”) [12]. An imbalance between oxidative stress and antioxidant systems might be triggered by toxic environmental exposures such as to heavy metals and xenobiotics. It is assumed that such an imbalance might lead to neuronal damage in genetically predisposed individuals [13–15]. Similar pathophysiological models have been discussed for schizophrenia [16]. Such theories for ASD have been supported by laboratory and postmortem findings of altered GSH concentrations in ASD patients. GSH is the brain’s dominant antioxidant, showing decreased blood levels of reduced GSH and increased concentrations of oxidized glutathione (GSSG) in the ASD group compared to controls on a meta-analytical level [14]. Postmortem studies found a disturbed redox ratio as a marker of oxidative stress in the cerebellum and temporal cortex [3, 17].

#### The antioxidant glutathione (GSH)

GSH is the main cellular free radical scavenger in the brain, playing a key role in protecting cells from exogenous and endogenous toxins, particularly in the brain [3, 14]. It is a tripeptide consisting of glutamate, cysteine, and glycine (L-c-glutamyl-L-cysteinylglycine). Cysteine is the rate-limiting amino acid for the synthesis of GSH [14]. It changes between the reduced monomeric (GSH) and oxidized dimeric forms (GSSG) in the scavenging process [16, 18]. The GSH/GSSG relation is an indicator of cellular redox status [19]. In the healthy brains, the ratio of GSH/GSSG is over 100 [20, 21] and GSSG is normally below MRS detection levels. GSH is essential for the synthesis and degradation of proteins and the formation of the deoxyribonucleotide precursors of DNA. It protects the cell against reactive oxygen compounds and conjugates with foreign compounds. It is also a cofactor in several other reactions [21].

### Magnetic resonance spectroscopy (MRS) as a tool for assessing GSH levels in vivo

MRS is a unique, non-invasive, and non-radioactive method of measuring different neurometabolites. For ASD research, MRS is of great interest because it is the only method for the non-invasive measurement of the main antioxidant, GSH, and of the major excitatory (glutamate, Glu) and inhibitory (γ-aminobutyric acid, GABA) transmitters in the human brain. In this study, we used the single-voxel proton MRS technique, which enables metabolite quantification in a predetermined volume of interest (VOI) with internal water reference scaling. Moreover, MRS is able to quantify additional neurometabolites, including creatine (Cre), which is a marker of the brain’s energy metabolism; choline (Cho) compounds or total-choline (t-Cho), which are markers of membrane/phospholipid turnover and white/gray matter differences; N-acetylaspartate (NAA), a marker of neuronal and axonal integrity; and myo-inositol (mI), an organic osmolyte and second messenger [21, 22].

#### Methodological MRS aspects

For the detection and quantification of GSH, various more or less sophisticated MRS methods have been applied. As apart from GSH, other important brain metabolites were to be detected and quantified in our study; we used short-TE PRESS (echo time-point-resolved spectroscopy) for good overall signal-to-noise ratio (SNR) and acceptable scan durations. Short-TE PRESS has been validated for GSH quantification with phantom experiments by others [23] and has been applied in clinical studies for voxel locations, which are similar to ours [24] or closer to the sinuses and thus even more challenging in terms of spectral quality [23]. MEGA-PRESS (Meshcher Garwood-PRESS), which has been established as a gold standard for GABA detection, has also been proposed for GSH editing. However, a good editing efficiency requires long echo times (TE ≈ 120 ms [25]), for which the rather short T2 of GSH (T2 = 67 ms at 4T [26]) gives rise to considerable signal loss compared to short-TE PRESS. 2D MRS methods such as JPRESS (J-resolved pointe-resolved spectroscopy) or COSY (correlation spectroscopy) have also been proposed for GSH detection. However, the 2D-fitting methods required for quantification of these spectra still lack the robustness of the established linear combination of a model spectra (LCModel) algorithm.

#### Previous MRS findings in the prefrontal cortex

Only one study analyzing GSH concentrations in ASD has been published to date. Durieux et al. [24] found no GSH signal differences between male ASD patients and age- and IQ-matched controls in the basal ganglia and the dorsomedial prefrontal cortex (DMPFC). Several schizophrenia studies have been performed with mixed results: In the largest study in first-episode schizophrenic patients, 22% higher medial temporal lobe GSH concentrations were found compared to the control group [27]. However, two other studies analyzing prefrontal regions showed no group differences between the schizophrenia and healthy control groups [28, 29]. Studies analyzing the established neurometabolites in ASD have mainly focused on the anterior cingulate cortex (ACC). Most of these studies found abnormal glutamate concentrations [9, 10, 30–32]. Cochran et al. [33] found higher glutamine (Gln) levels and lower GABA/Cre levels in ASD, supporting the idea of an excitatory (Glu)/inhibitory (GABA) imbalance hypothesis. Anterior cingulate NAA, t-Cho, Cre, and mI signals were also found to be altered in some earlier studies [9, 10, 31, 34–37]. In the left dorsolateral prefrontal cortex (DLPFC), low NAA/Cre levels were found in the study by Fujii and colleagues [36], but no significant differences were found in two other studies [34, 38].

### Rationale of our study

The main aim of this study was to compare GSH signals in high-functioning ASD patients with normal and above average intelligence quotients (IQ >90) and those in healthy controls. We excluded patients with subnormal IQs because of their possible link to syndromal and secondary forms of autism. We decided to analyze the dorsal ACC because of the recently reported neurochemical abnormalities in this region [9, 10]. In addition, we analyzed the DLPFC, which is an important part of the dorsolateral prefrontal circuits [39]. Alterations in these circuits lead to executive dysfunction [39]. The DLPFC is also associated with deficits in theory of mind [38, 40]. Neurochemical alterations in the DLPFC were described earlier [9]. Based on the evidence from several laboratory and postmortem studies [3, 14, 17], we hypothesized that we would find decreased GSH concentrations in the dACC and DLPFC of ASD patients. Moreover, the study examined Cre, t-Cho, Glx = Glu + Gln, NAA, and mI signals. On the basis of earlier studies suggesting an excitatory-inhibitory imbalance mechanism [9, 10], we hypothesized that we would find altered Glx levels in the ACC.

## Methods

All patients were recruited at the Freiburg Center for the Diagnosis and Treatment of Autism (University Center for Autism Spectrum, Universitäres Zentrum Autismus Spektrum Freiburg, UZAS; www.uniklinik-freiburg.de/psych/live/patientenversorgung/schwerpunkte/schwerpunkt-asperger.html).

### Patient assessment

The diagnostic process followed the National Institute for Health and Clinical Excellence (NICE) guidelines for adult autism (http://guidance.nice.org.uk/CG142/NICEGuidance/pdf/English). Only patients fulfilling the ICD-10 F84.5 and DSM-IV 299.00 criteria were included. The structured diagnostic procedure was performed by a multiprofessional diagnostic team with three experienced senior consultant psychiatrists and two fully qualified senior psychologists. Anamnesis was conducted over several sessions and included questioning of caregivers or relatives (parents, siblings, partners, etc.) and behavioral observations. The diagnosis was made by all persons involved in the diagnostic process, incorporating at least two experienced consultant psychiatrists or psychologists. Psychometric assessments using the autism-spectrum quotient (AQ) [41], empathy quotient (EQ) [42], Australian Scale of Asperger’s Syndrome [43], Social Responsiveness Scale [44], Bermond-Vorst Alexithymia Questionnaire [45], and Adult Asperger Assessment [46] were included in the diagnostic process. The Wender Utah Rating Scale (WURS-k) [47] for ADHD symptoms and the Beck Depression Inventory score (BDI) [48] for depressiveness were collected to assess the most frequent comorbidity and to control for influences of their symptoms on metabolic signals. General crystalline intelligence was assessed using the multiple-choice word test B (MWT-B) [49]. In unclear cases, the Autism Diagnostic Interview-Revised [50], the autism diagnostic observation schedule-generic [51], and/or behavioral assessments as an in-patient were additionally included. To avoid a heterogeneous study sample, we excluded secondary and obviously syndromal forms of ASD and only included patients with normal or above average IQ.

### Healthy control group

The control group was comprised of age-, IQ-, and gender-matched healthy subjects. Controls with relevant medical, psychiatric, or neurological diseases were excluded from the study. All controls were assessed with the AQ, EQ, WURS-k, BDI, and MWT-B questionnaires. AQ scores > 30, EQ scores ≤ 30, WURS-k scores > 30, BDI scores > 18, and IQ scores < 90 led to exclusion from the study.

### MRS procedure

**Fig. 1:**
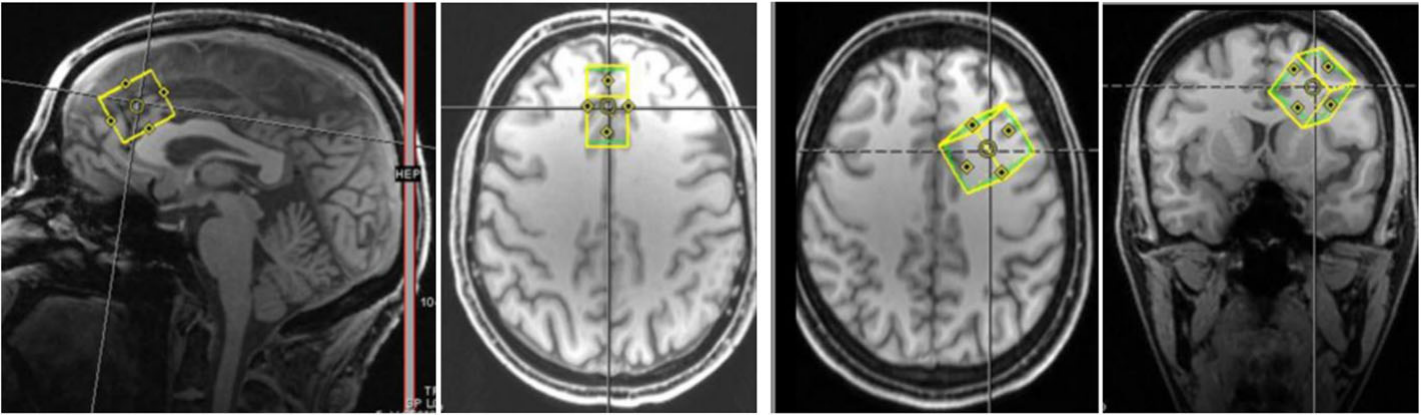
Voxel localization in the dorsal anterior cingulate cortex in both sides (*two images on the left*) and in the left dorsolateral prefrontal cortex (*two images on the right*)

**Fig. 2:**
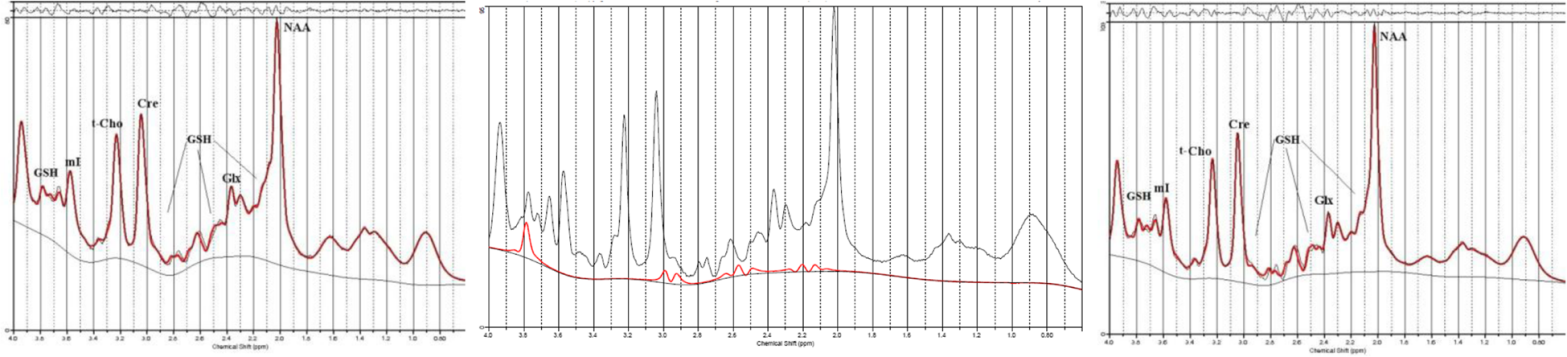
MR-spectrum from the dorsal anterior cingulate cortex (*left*) and in the left dorsolateral prefrontal cortex (dACC; *right*). In the *middle*, we present glutathione fitting in a patient spectrum from the dACC. *GSH* glutathione, *mI* myo-inositol, *t-Cho* phosphorylcholine + glycerylphosphorylcholine, *Cre* creatine, *Glx* glutamate + glutamine, *NAA* N-acetylaspartate

All measurements were performed at the University of Freiburg on a 3 Tesla Siemens Magnetom TIM Trio system (Erlangen, Germany). For signal reception, a 32-channel head coil was used. First, a standard magnetization-prepared rapid gradient echo (MPRAGE) T1-weighted anatomical scan was recorded with the following parameters: field of view (FOV) = 256 × 256 mm^2^, repetition time (TR) = 2200 ms, TE = 4.11 ms, flip angle = 12°, and voxel size = 1 × 1 × 1 mm^3^. Spectroscopic measurements were performed in the dorsal anterior cingulate cortex (30 × 20 × 25 mm; 15.0 mL) and in the left dorsolateral prefrontal cortex (25 × 25 × 25 mm; 15.6 mL) using a PRESS sequence with a TR of 1500 ms, a TE of 30 ms, and a number of slices of 256. The PRESS sequence used in this work contained Hamming-filtered sinc pulses with a bandwidth of 3365 Hz for excitation and Mao pulses with a bandwidth of 1153 Hz for refocusing. The signal readout consisted of 1024 sampling points covering a bandwidth of 1200 Hz. For metabolite quantification with the internal water reference method, a water spectrum was acquired using the same protocol as for the acquisition of the actual spectra, but the radio frequency pulses for water suppression were switched off, and the number of spectral averages was reduced to 16 [52]. For spectral fitting and quantification, the well-established and validated LCModel algorithm was applied (http://s-provencher.com/lcmodel.shtml). The metabolite basis set for LCModel quantification was simulated numerically with Matlab, using chemical shifts and coupling constants derived from the literature, and fully shaped RF waveforms of the slice-selective refocusing pulses as employed by the scanner [53]. For spectroscopic analyses, only spectra with Cramér-Rao lower bounds (CRLBs) smaller than 20% for the main metabolites were included [54, 55]. Finally, the 3D MPRAGE datasets were segmented into gray matter (GM), white matter (WM), and cerebrospinal fluid (CSF), using a unified segmentation approach [56] implemented in Statistical Parametric Mapping, Version 8 (SPM8; www.fil.ion.ucl.ac.uk/spm/software/spm8). For each VOI, the partial volumes of GM, WM, and CSF were computed from this segmentation. These partial volumes were used for estimation of the water content in the VOI, which was needed for quantification and for metabolite concentration correction, assuming that the measured brain metabolites are only contained in GM and WM, but not in CSF [9, 10, 56, 57] (Figs. 1 and 2).

### Study sample

Table 1 gives an overview of the study sample. In the ASD group, only one patient had to be excluded due to technical reasons; in the control sample, we excluded five subjects because of suprathreshold psychometric findings and one control due to technical reasons.

**Table 1:** Autism spectrum disorder and healthy control samples with reasons for exclusion

	Autism spectrum disorder	Controls
Included subjects	25	24
Technical reasons for further exclusion	1 × bad spectral quality	1 × bad spectral quality
Post hoc information about psychometric exclusion criteria	0	1× BDI-score >18, 1× WURS-k-score >30, 2× EQ-score ≤30, 1× IQ <90
Number of spectra for statistical analysis	24	18

*BDI* Beck Depression Inventory score, *WURS-k* Wender Utah Rating Scale, *EQ* empathy quotient, *IQ* intelligence quotient

### Statistical analyses

Spectroscopic, demographic, and psychometric data were transferred to a Statistical Package for the Social Sciences (SPSS inc., Stanford USA) database. For group comparisons of continuous variables (age, nicotine consumption, psychometric scores), we performed two-sided independent sample *t* tests. For group comparisons of gender, we calculated Pearson’s two-sided chi squared test. The neurometabolite concentrations in the dACC and DLPFC of the ASD and control groups were compared using a multivariate analysis of covariance (MANCOVA) and employing a general linear model. The factor group was chosen as the fixed factor and the neurometabolites (GSH, Cre, t-Cho, Glx, NAA, and mI) as dependent variables. No covariates were included in the first MANCOVA. The possible confounding factors of age [58], IQ [59], gender [60, 61], and nicotine consumption [62, 63] did not differ between the groups. Therefore, we did not include these variables as covariates in the main analyses (as we did in earlier studies [57]). However, we performed another analysis with age, IQ, and nicotine consumption as covariates to exclude effects due to within-subject variations in these parameters. To test for an overall group effect across all six neurometabolites, a multivariate Wilks’ lambda test was used in both cases. When comparing medicated and unmedicated ASD patients, we found significant differences in the gender ratio; therefore, we also included gender (besides group [medicated vs. unmedicated]) as a fixed variable in the respective MANCOVA. Correlation analyses were performed using the Pearson correlation coefficient. To test for the influence of age on the correlation between GSH and IQ, we performed a partial correlation, adjusted for age. For overall and single-group differences in neurometabolite signals, the level of significance was corrected for multiple tests using the Bonferroni approach (*p* < 0.025 due to performing the measurements in two independent regions). The same significance level of *p* < 0.025 was used for the correlation analyses (because of the two measured regions), and for all other calculations, a *p* value <0.05 served as the criterion of significance.

## Results

### Demographic and psychometric data

The ASD and control groups did not differ significantly in age, IQ, nicotine consumption, and gender. The age range varied from 25 to 57 years in the ASD group and from 24 to 60 years in the control sample. Table 2 also gives detailed information about levels of psychometric scores, as well as education and occupational situation for all participants.

**Table 2:** Demographic and psychometric information for the autism spectrum disorder and control groups

	ASD (*n* = 24)	Controls (*n* = 18)	Statistics
Age	40.54 ± 10.279	37.17 ± 10.983	*p* = 0.313
Intelligence quotient	114.29 ± 15.213	113.00 ± 13.920	*p* = 0.779
Gender	12 m:12 f	8 m:10 f	*p* = 0.721
Nicotine	2.292 ± 5.894	2.222 ± 6.691	*p* = 0.972
Autism-spectrum quotient	39.08 ± 6.386	12.94 ± 5.504	*p* < 0.001
Empathy quotient	17.38 ± 9.523	47.94 ± 9.710	*p* < 0.001
Wender Utah Rating Scale	33.75 ± 16.690	8.72 ± 7.954	*p* < 0.001
Beck Depression Inventory score	17.25 ± 12.365	3.22 ± 4.236	*p* < 0.001
Level of education	Low school degree, 1 (4.2%)	Low school degree, 0 (0%)	
	Medium school degree, 3 (12.5%)	Medium school degree, 3 (16.7%)	
	High school degree, 9 (37.5%)	High school degree, 6 (33.3%)	
	University degree, 9 (37.5%)	University degree, 9 (50%)	
	Other, 2 (8.3%)	Other, 0 (0%)	
Occupational situation	Full-time employed, 13 (54.2%)	Full-time employed, 7 (38.9%)	
	Part-time employment, 4 (16.7%)	Part-time employment, 4 (22.2%)	
	In vocational training/studying, 1 (4.2%)	In vocational training/studying, 7 (38.9%)	
	Pension, 1 (4.2%)	Pension, 0 (0%)	
	Unemployed, 4 (16.7%)	Unemployed, 0 (0%)	
	Other, 1 (4.2%)	Other, 0 (0%)	
Psychiatric medication (in the last 3 months)	No, 11	No, 18	
	AD, 6	AD, 0	
	NL, 1	NL, 0	
	AD + NL, 5	AD + NL, 0	
	AD + MPH, 1	AD + MPH, 0	

*ASD* autism spectrum disorder, *IQ* intelligence quotient, *AD* antidepressants, *NL* neuroleptics, *MPH* methylphenidate

For references, see text

### MRS results

We acquired robust data with high SNR (40.452 ± 27.974 for the ACC VOI and 39.0 ± 21.462 for the DLPFC voxel) and low average CRLBs for GSH (6.4% for the ACC voxel and 7.0% for the DLPFC voxel). Using a multivariate analysis of covariance, significant group differences were found neither in the dACC (Wilks’ lambda test without covariates *p* = 0.429, with covariates *p* = 0.564) nor in the DLPFC (without covariates *p* = 0.288, with covariates *p* = 0.262; Table 3).

**Table 3:** Neurometabolic results for both analyzed regions (in mM)

	Dorsal anterior cingulate cortex		MANCOVA without covariates (Wilks’ lambda test *p* = 0.429)	MANCOVA with covariates^a^ (Wilks’ lambda test *p* = 0.564)	Dorsolateral prefrontal cortex		MANCOVA without covariates (Wilks’ lambda test *p* = 0.288)	MANCOVA with covariates^a^ (Wilks’ lambda test *p* = 0.262)
	ASD (*n* = 24)	Controls (*n* = 18)			ASD (*n* = 24)	Controls (*n* = 18)		
GSH	1.7939	1.9810	*p* = 0.076	*p* = 0.147	1.6017	1.6247	*p* = 0.706	*p* = 0.655
	0.37068	0.26364			0.18921	0.19985		
Cre	7.4948	7.9657	*p* = 0.048	*p* = 0.082	6.9109	6.6330	*p* = 0.013	*p* = 0.020
	0.91646	0.39453			0.33509	0.35355		
t-Cho	1.8900	1.9926	*p* = 0.213	*p* = 0.200	1.8507	1.8289	*p* = 0.757	*p* = 0.984
	0.27320	0.24132			0.18029	0.27268		
Glx	11.7396	12.5834	*p* = 0.142	*p* = 0.272	9.4585	9.2360	*p* = 0.396	*p* = 0.094
	2.14178	1.21151			0.97482	0.58407		
NAA	8.1840	8.8939	*p* = 0.168	*p* = 0.319	8.8931	8.7972	*p* = 0.661	*p* = 0.423
	1.82291	1.29632			0.75962	0.59745		
mI	5.8211	6.3095	*p* = 0.018	*p* = 0.036	5.0549	4.9064	*p* = 0.344	*p* = 0.456
	0.73532	0.46517			0.50464	0.48836		

*ASD* autism spectrum disorder, *MANCOVA* multivariate analysis of covariance, *p p* value to test for differences between groups, *GSH* glutathione, *Cre* creatine, t-Cho phosphorylcholine + glycerylphosphorylcholine, *Glx* glutamate + glutamine, *NAA* N-acetylaspartate, *mI*, myo-inositol

^a^Age, intelligence quotient, and nicotine consumption

#### GSH

On the level of single metabolite concentrations, there were tendencies for decreased anterior cingulate GSH levels in the ASD group (ASD group, 1.79 mM ± 0.37; control group, 1.98 mM ± 0.26; *p* = 0.076) that were not stable in the MANCOVA analyses with potential influencing factors as covariates (*p* = 0.147).

#### Other neurometabolites

The anterior cingulate mI signals were also lower in the ASD group (5.82 mM ± 0.74 to 6.31 mM ± 0.47; *p* = 0.018). Cre concentrations in the ASD sample were lower in the dACC region (7.49 ± 0.92 to 7.97 ± 0.39; *p* = 0.048) and higher in the DLPFC VOI (6.91 ± 0.34 to 6.63 ± 0.35; *p* = 0.013).

### Analysis of dimensional associations

#### GSH

The correlation analysis for the ASD sample (*N* = 24) in the dACC VOI revealed a significant negative correlation between the GSH signal and IQ (*r* = −0.549; *p* = 0.005, *N* = 24) and age (*r* = −0.567, *p* = 0.004, *N* = 24); however, a partial correlation for GSH and IQ—corrected for age—was not significant (*r* = −0.290; *df* = 21; *p* = 0.179, *N* = 24).

#### Other neurometabolites

In addition, we found a highly significant negative correlation between age and the Glx signal in the DLFC (*r* = −0.730, *p* = <0001, *N* = 24) (Table 4).

**Table 4:** Correlation analyses for the autism spectrum disorder group (a *p* value of <0.025 served as the criterion of significance)

	AQ	EQ	WURS-k	BDI	IQ	Age
Anterior cingulate cortex (*n* = 24)						
GSH	0.077	−0.193	0.287	−0.006	*−0.549*	*−0.567*
	0.721	0.366	0.174	0.978	*0.005*	*0.004*
Cre	0.183	−0.373	−0.103	−0.082	−0.329	−0.310
	0.393	0.072	0.632	0.702	0.117	0.140
t-Cho	0.286	−0.268	0.063	0.010	−0.106	−0.195
	0.175	0.206	0.770	0.964	0.622	0.360
Glx	0.301	−0.394	0.104	−0.019	−0.370	−0.346
	0.152	0.057	0.628	0.931	0.075	0.098
NAA	0.363	−0.399	−0.007	−0.102	−0.297	−0.310
	0.081	0.053	0.974	0.634	0.159	0.140
mI	−0.011	−0.040	−0.013	−0.057	−0.289	−0.339
	0.959	0.853	0.952	0.793	0.171	0.105
Dorsolateral prefrontal cortex (*n* = 24)						
GSH	−0.014	−0.058	0.126	0.154	−0.020	−0.166
	0.950	0.790	0.556	0.471	0.927	0.438
Cre	0.042	−0.176	−0.287	−0.283	−0.136	−0.115
	0.847	0.410	0.173	0.180	0.527	0.593
t-Cho	−0.226	0.443	−0.010	−0.027	0.434	0.097
	0.289	0.030	0.964	0.901	0.034	0.653
Glx	−0.079	0.162	0.312	−0.179	−0.454	*−0.730*
	0.713	0.450	0.138	0.402	0.026	*0.000*
NAA	−0.038	−0.297	0.183	0.033	−0.341	−0.391
	0.861	0.159	0.391	0.880	0.103	0.059
mI	−0.205	0.310	0.004	−0.349	0.005	0.035
	0.336	0.141	0.987	0.094	0.982	0.870

Presented are the Pearson correlation coefficients (above) and *p* values (below)

Significant results are depicted in italics

*AQ* autism quotient, *EQ* empathy quotient, *WURS* Wender Utah Rating scale, *BDI* Beck Depression Inventory score, *IQ* intelligence quotient, *GSH* glutathione, *Cre* creatine, *t-Cho* phosphorylcholine + glycerylphosphorylcholine, *Glx* glutamate + glutamine, *NAA* N-acetylaspartate, *mI* myo-inositol (for references, see text)

#### Medication effects

Finally, we compared medicated (*n* = 13) and unmedicated ASD patients (*n* = 11). Both groups showed no significant differences in the possibly confounding factors of age, IQ, and nicotine consumption. Since they differed in gender ratio, we performed a MANCOVA with the fixed variables of medication and gender. For both VOIs, we found no significant group differences (Wilks’ lambda test, *p* = 0.891 for the dACC and *p* = 0.484 for the DLPFC).

## Discussion

The main finding of this study is the absence of significant group differences in the dACC or in the DLPFC with respect to GSH. Therefore, we were not able to confirm our working hypothesis regarding decreased GSH concentrations in the ASD group.

### Limitations

First, potential limitations should be considered. The diagnostic procedure was performed by an experienced, multiprofessional diagnostic team following the NICE guidelines for adult autism. To avoid a heterogeneous study sample, we included only patients with normal or above average IQs. Therefore, our cohort is not representative for low-functioning autism. The advanced age of our patients should also be considered. Because ASD is a neurodevelopmental disorder, the GSH metabolism could have normalized over the years. The sample size is comparable with other studies analyzing GSH metabolism [24, 27]. One limitation is that we analyzed patients with and without medication. Therefore, our results might be influenced by medication-induced oxidative stress [64], and further studies should analyze unmedicated patients. However, in our sample, we did not find any significant differences in neurometabolite concentrations between medicated and non-medicated patients. We therefore do not assume a relevant medication effect in our cohort.

For MRS measurements, we used the well-established single-voxel 1H-MRS method (applying short-TE PRESS). The accuracy of GSH detection with such standard MRS methods is a strongly disputed topic in the MRS community (see “Background” section). However, standard MRS sequences (PRESS and STEAM) have previously been applied in a number of other GSH studies in humans, and to date, there is no clear evidence of better in vivo GSH quantification accuracy with different editing methods such as MEGA-PRESS [23, 27, 65–67]. The measurement of GSH levels is complicated due to significant resonance overlap with other metabolites [21]. By using a 32-channel head coil at a field strength of 3 T and the manual shimming procedure, we obtained robust data with high SNR and low average CRLBs for GSH. We were only able to measure GSH signals. The GSH/GSSG ratio would have been a better indicator of cellular redox status; however, the GSSG concentration was below the MRS detection limit [68]. We used the established and stable SVS method, which allows only the measurement of preselected VOIs. Therefore, we were only able to measure the concentrations of neurometabolites of interest in preselected VOIs (dACC and DLPFC). Data exclusion was performed based on the CRLBs determined by LCModel (rejection for CRLBs >20% for the main metabolites). In psychiatric MRS studies, metabolite concentrations are often reported as ratios to the Cre concentration, which is considered to be relatively unaffected by most pathological conditions. In our study, we performed metabolite quantification with internal water reference scaling to avoid bias arising from varying levels of Cre. In fact, we did find a trend of decreased Cre signals in the dACC and a tendency for Cre signals to be increased in the DLPFC of patients, suggesting that metabolite quantification is more robust than calculating ratios over Cre, as in other ASD studies. The LCModel software allowed all spectra to be treated the same.

### Interpretation of our findings in the context of earlier studies

This was the first study analyzing anterior cingulate GSH signals, and we only found a tendency for decreased GSH concentrations. The only currently available study analyzing GSH concentrations in ASD found no differences in the left basal ganglia and the left DMPFC [24]. Therefore, our observation of normal GSH concentrations in the DLPFC is compatible with the negative findings of Durieux et al. [24] in the DMPFC. In the region of the left DLPFC, two other studies also failed to detect any metabolic changes [34, 38]. We found decreased levels of Glx in the dACC, however, without reaching a level of significance, in contrast to earlier studies in the pregenual ACC, where a significant Glx decrease was observed [9, 10, 30]. In other studies, increased Glx levels were found in the ACC [31, 32], suggesting fluctuating levels of glutamate. Similar patterns of findings are reported in other neuropsychiatric conditions like epilepsy or anti-N-methyl-D-aspartate-receptor encephalitis [9, 69]. GSH and Glx metabolisms are closely related: GSH is synthesized from Glu, cysteine, and glycine [21]. Glutathione is a physiologic reservoir of neuronal Glu [70]. Glutamatergic metabolism seems to play an important role in ASD, possibly related to a cybernetic imbalance between neuronal excitation and inhibition [9, 10]. Glu is the major excitatory neurotransmitter [71], and Gln is its precursor and storage form in astrocytes [53]. Low-plasma Gln may deplete GSH, because Gln provides Glu to synthesize glutathione; GSH depletion may deplete Gln for the same reason [12]. In their ASD meta-analysis, Frustraci et al. found decreased blood levels of reduced glutathione (27%) and increased concentrations of oxidized glutathione (GSSG; 45%) relative to controls [14], suggesting a role of redox imbalance in the pathogenesis of ASD. Postmortem studies examining the temporal cortex and the cerebellum showed decreased GSH/GSSG ratios as a marker for oxidative stress [3, 17]. In the current study, we were not able to detect significant group differences in GSH signals between patient and control groups in the dACC and the DLPFC. Our results might be influenced by several factors such as medication (see “Limitations” section). Therefore, the examination of further regions, especially the temporal cortex and the cerebellum (because of the earlier described alterations in postmortem studies) in medication-free ASD patients should be performed. In addition, we found a trend for reduced mI concentrations in the dACC. Reduced mI levels may be associated with abnormal developmental processes via abnormal astrocyte density or altered neuronal growth [72], as shown in children with ASD [72, 73]. However, earlier findings are inconsistent [35, 72]. The trend for alterations in the Cre signals might be associated with brain-region-specific altered energy metabolism [22]. Decreased Cre signals, as observed in the dACC, are in line with the research of Bejjani et al. [31]. A tendency for higher Cre concentrations in the ASD group has not been described previously.

The dimensional analyses showed dACC GSH signal to be linked to IQ and age but not to autistic symptoms. We found a negative correlation between the GSH signals in the dACC and the IQ scores; the association between high IQ figures and low GSH levels is contrary to earlier research, which assumed impairment in cognitive function with reducing GSH levels [74]. Moreover, dACC GSH signals in the ASD collective correlated negatively with age; earlier studies also described an age-dependent decline of GSH concentrations [21]. A partial correlation between GSH and IQ, corrected for age, was not significant.

## Conclusions

We were not able to detect significant alteration in GSH levels in the dACC and DLPFC of ASD patients. Significantly decreased GSH levels were earlier reported in blood and postmortem studies of the temporal cortex and the cerebellum [17]. Further MRS studies should focus on the temporal cortex and the cerebellum in medication-free ASD patients. Moreover, we suggest a combined blood and MRS measurement of GSH signals. Whereas the MRS measurements allow regional metabolic investigations in certain brain regions, blood analyses would allow the determination of the whole redox system by also measuring GSSG signals. Moreover, CSF measurements of the GSH/GSSG ratios could provide additional information [75]. The topic of antioxidants is of high public interest and important for patients because of the possible treatment options with supplemental antioxidants (e.g., with N-acetylcysteine, a GSH precursor) [18].

## Abbreviations

ADHD: Attention deficit hyperactivity disorder
AQ: Autism-spectrum quotient
ASD: Autism spectrum disorder
BDI: Beck Depression Inventory score
Cre: Creatine
CRLBs: Cramér-Rao lower bounds
CSF: Cerebrospinal fluid
dACC: Dorsal anterior cingulated
DLPFC: Dorsolateral prefrontal cortex
DMPFC: Dorsomedial prefrontal cortex
EQ: Empathy quotient
FOV: Field of view
GABA: γ-Aminobutyric acid
Gln: Glutamine
Glu: Glutamate
GM: Gray matter
GSH: Glutathione
GSSG: Glutathione disulfide
IQ: Intelligence quotients
LCModel: Linear combination of model spectra
MANCOVA: Multivariate general linear model
mI: myo-Inositol
MPRAGE: Magnetization-prepared rapid gradient echo
MRS: Magnetic resonance spectroscopy
MWT-B: Multiple-choice word test B
NAA: N-Acetylaspartate
NICE: National Institute for Health and Clinical Excellence
PRESS: Point-resolved spectroscopy
SPM: Statistical Parametric Mapping
SVS: Single-voxel spectroscopy
T: Tesla
t-Cho: Total-choline
TE: Echo time
TR: Repetition time
VOI: Volume of interest
WM: White matter
WURS: Wender Utah Rating Scale
